# The difficulty of aligning intrinsically disordered protein sequences as assessed by conservation and phylogeny

**DOI:** 10.1371/journal.pone.0288388

**Published:** 2023-07-13

**Authors:** Andrew C. Riley, Daniel A. Ashlock, Steffen P. Graether

**Affiliations:** 1 Graduate Program in Bioinformatics, University of Guelph, Guelph, Ontario, Canada; 2 Department of Molecular and Cellular Biology, University of Guelph, Guelph, Ontario, Canada; 3 Department of Mathematics & Statistics, University of Guelph, Guelph, Ontario, Canada; Naval Postgraduate School, UNITED STATES

## Abstract

Intrinsically disordered proteins (IDPs) are proteins that lack a stable 3D structure but maintain a biological function. It has been frequently suggested that IDPs are difficult to align because they tend to have fewer conserved residues compared to ordered proteins, but to our knowledge this has never been directly tested. To compare the alignments of ordered proteins to IDPs, their multiple sequence alignments (MSAs) were assessed using two different methods. The first compared the similarity between MSAs produced using the same sequences but created with Clustal Omega, MAFFT, and MUSCLE. The second assessed MSAs based on how well they recapitulated the species tree. These two methods measure the “correctness” of an MSA with two different approaches; the first method measures consistency while the second measures the underlying phylogenetic signal. Proteins that contained both regions of disorder and order were analyzed along with proteins that were fully disordered and fully ordered, using nucleotide, codon and peptide sequence alignments. We observed that IDPs had less similar MSAs than ordered proteins, which is most likely linked to the lower sequence conservation in IDPs. However, comparisons of tree distances found that trees from the ordered sequence MSAs were not significantly closer to the species tree than those inferred from disordered sequence MSAs. Our results show that it is correct to say that IDPs are difficult to align on the basis of MSA consistency, but that this does not equate with alignments being of poor quality when assessed by their ability to correctly infer a species tree.

## Introduction

The link between structure and function is a crucial concept in studying and understanding the roles of proteins. However, greater attention is being given to proteins and protein regions that lack a stable three-dimensional (3D) structure and what functional roles they may play. These are known as intrinsically disordered proteins (IDPs), and by definition contain a continuous stretch of at least 30 disordered residues [1]. IDPs can be fully disordered, or they can have stretches of disordered residues, known as intrinsically disordered regions (IDRs). IDPs are highly flexible due to the lack of a stable secondary and tertiary structure, which is due to their primary structure being enriched with polar and charged amino acids while hydrophobic residues appear less frequently. The lack of a stable 3D structure means IDPs do not face the same evolutionary constraints as structured proteins, since they do not need to maintain an ordered structure to preserve their function [2]. This has been shown in various studies, where it was found that the majority of IDPs undergo a higher rate of amino acid substitution when compared to ordered proteins and regions [3,4]. It has also been observed that insertions and deletions (indels) occur more frequently in disordered regions, likely due to the lower selective pressure in these regions [5]. This combination of higher substitution rates and more frequent indels may indicate that some IDPs are undergoing rapid evolution, which could result in a lower sequence identity between orthologues [6].

Due to this lower sequence conservation and more frequent indels, the unverified thought is that IDPs are challenging to align [6–9]. Alignments are important tools for identifying and comparing similar sequences, and can provide insight into a protein’s function by identifying key, conserved residues. Alignments are also commonly used to build phylogenetic trees to perform evolutionary analyses; if an alignment is not accurate it can result in a less accurate phylogenetic tree that cannot be used for a reliable evolutionary analysis [10]. In a study by Mahani *et al*. on the Myc family of proteins, which are IDPs, a phylogenetic tree was created to better understand the relationship between the different families [11]. However, they found that the tree they built from an MSA lacked sufficient bootstrap support to reliably establish connections between all of the families. In our research on dehydration-induced proteins (dehydrins), which are fully disordered proteins that are found in plants, we were similarly trying to understand how different groups of dehydrins are related using phylogenetic trees derived from MSAs [12]. As with the Mahani *et al*. (2013) study [11], we found the phylogenetic tree bootstrap support too low to reliably establish connections between the groups. Instead, we relied on synteny to verify relationships, but this could only be used with closely related dehydrins. In both studies, it was suspected that the poorly supported phylogenetic trees were the result of low quality MSAs, in part due to the disordered nature of the proteins being aligned.

Unfortunately assessing the quality of MSAs is a challenging task in itself, and disorder makes it more difficult. The “gold standard” for MSA assessment is consensus with a structural alignment [8,13]. If regions within an MSA agree with the alignment of 3D-structures of the same proteins, there is strong support that these regions in the sequences are truly homologous. However, this method is of little to no use when assessing disordered proteins since they lack the 3D-structure required for the comparison. Alternative approaches are therefore needed to assess the quality of MSAs created from IDP sequences. Consistency-based methods can be applied to IDP MSAs and do not rely on any existing biological information. These methods do rely on the assumption that if different aligners are in agreement on the MSA of a set of sequences, it is more likely that this MSA is the correct one [14–16]. Another approach to MSA evaluation is phylogeny-based, where the assumption made is that given a known phylogenetic tree, the closer the phylogenetic tree created from an MSA is to the known tree, the more accurate the alignment [17]. Species-tree discordance uses the species tree as the known tree and can be applied to sequences from orthologous genes [17]. This method assumes that the divergence between orthologous genes should match speciation events, and therefore the phylogenetic signal from the MSA of the orthologous genes should create a tree similar to the species tree.

In this work, we assess and compare the MSAs of IDPs to ordered proteins using two approaches. The first approach is a consistency-based method, where MSA quality is determined by the similarity of MSAs created using several different alignment methods. The second approach is a phylogeny-based method similar to species tree discordance [17], where phylogenetic trees of protein from a large number of mammalian taxa are compared to the mammalian species tree. The quality of the MSAs is determined by the proximity of their phylogenetic tree to the species tree. By assessing how well IDPs are aligned, we can better understand how and when alignments should be applied to this important group of proteins.

## Materials and methods

### Sequence and species tree collection

Three sets of proteins were collected to compare MSAs of disordered sequences to MSAs of ordered sequences: proteins that contain both regions of order and disorder (which are called “mixed proteins” throughout the text), fully ordered proteins, and fully disordered proteins. The use of mixed proteins allows for a direct comparison of the disordered region to the ordered region within the same protein, which helps to minimize the impact of divergence due to evolution and inconsistencies, such as horizontal gene transfer or the inclusion of paralogues. Although the sequence content in the disordered and ordered regions will differ, they are collected from the same sequences, and therefore would be equally impacted.

While the mixed proteins allow for comparisons of disorder and order on an individual protein, the fully ordered and disordered proteins must be compared as groups of proteins. Therefore, comparisons of the fully ordered and disordered proteins would need to representative of all fully ordered and disordered proteins for a meaningful comparison. However, collecting and analyzing a sufficient number of proteins to be representative of both groups as a whole is computationally intractable using the approach described here. The comparison of the fully ordered and disordered proteins here is therefore intended to establish if the results obtained from these comparisons are consistent with observations made with the mixed proteins.

The first step was to select the initial sequence, which was to be used as the query sequence to identify orthologues in a number of mammalian taxa. Mammalian protein sequences were selected because the mammalian phylogenetic tree, although not fully resolved, has been well studied [18–20]. Additionally, there is a considerable amount of mammalian sequence data available when compared to other taxonomic groups. On DisProt 8.0, a search of *Mammalia* results in 9 times more entries than a search for *Viridiplantae*, a more ancient taxonomic group (Retrieved 02/06/22) [21,22].

Mixed proteins were identified through a multi-step procedure. First, the DisProt 8.0 database was manually searched for sequences that were not fully disordered [21,22]. Next, proteins that had contiguous disordered and ordered regions >30 residues long were collected. Disordered regions were identified as the longest DisProt consensus fragment while the ordered regions were identified as those that had the longest Gene3D domain [23]. If there was overlap between disordered and ordered regions, the overlapped region was excluded from analysis. Lastly, sequences where the combined length of the extracted disordered and ordered regions was <50% of the whole sequence were rejected. The DisProt 8.0 database was also used to collect the fully disordered proteins, where only sequences with a disorder content of 100% were retained. The UniProt Knowledgebase was used to collect the initial fully ordered proteins [24], where only sequences of well-studied proteins with experimentally determined structures were retained. For consistency, the same number of fully ordered sequences was collected as the number of fully disordered sequences.

The same method described above was used to collect a set of mammalian sequences for every initial protein sequence. A BLASTP search of the NCBI database was performed using the initial sequence to search for homologous ones [25,26]. Default settings were used (*E*-value threshold = 0.05, Word size = 6, Matrix = BLOSUM62, Gap existence = 11, Gap extension = 1); however, the search was limited to sequences from mammals and the number of hits was increased to 5000. Sequences that were not reference sequences (i.e. those without an NP or XP prefix) [27], contained ambiguous amino acids, or were labelled as ‘like’, ‘low quality’, or ‘partial sequences’ were rejected. For cases where there were multiple sequence hits within one species, the collected sequences were compared to the initial sequence by a pairwise alignment using the pairwise2 module in Biopython [28], and the sequence with the highest identity (i.e. highest proportion of exact matches to the query sequence) to the initial sequence was collected for each species. In order to check for paralogues, a reverse BLASTP was performed against the organism of the initial sequence. If the top hit was the initial sequence in the source organism, the sequence was kept. In the case of melanophilin, prothymosin α, and β-casein, a match to an alternative isoform during the reverse BLASTP was also accepted. For melanophilin and prothymosin α, only 25 and 33 sequences were initially collected after the reverse BLASTP. Both had alternative isoforms with an identity greater than 99% to the initial sequence, so matches to these isoforms were accepted. In the case of β-casein, the sequence with the highest identity match to the initial sequence had a higher match to a different isoform. Matches to this isoform were also accepted; the isoform had a 96% identity to the initial sequence. In the case of hexokinase 1 and L-lactate dehydrogenase A chain, several collected sequences were rejected because it was observed that small clades of unrelated species were present near the outgroup in both of their phylogenetic trees. Upon further investigation, it was found that these sequences had the lowest identity to the initial sequence and were likely paralogues. After establishing the complete set of sequences, the DNA sequences were collected using Batch Entrez [29].

Outgroup sequences were also collected for every protein. A BLASTP search was performed on *Gallus gallus* (chicken) using every initial protein, after which a reverse BLASTP was then performed on the top reference sequence hit (defined as the sequence with the top BLASTP score). If a sequence from *G*. *gallus* was not available in the first BLASTP search, or was not recovered as the top hit after the reverse BLASTP, the sequence from *Ornithorhynchus anatinus* (platypus) was used instead as an outgroup instead. In the case of β-casein and involucrin, no non-placental mammal sequences were identified, so *Loxodonta africana* (African bush elephant) was used as the outgroup. For these two cases, other sequences in the same clade in the species tree (Afrotheria and Xenarthra) were removed so that the outgroup would consist of only one leaf. The collected fully disordered and fully ordered protein sequences were run through SignalP-5.0 [30], and any detected signal peptides were removed.

A list of all species present was created for each protein used. This list was then submitted to TimeTree to infer the species phylogenetic tree [19]. If a species did not appear in TimeTree, its sequence was removed from the analysis.

### Multiple Sequence Alignments (MSAs)

**Fig 1:**
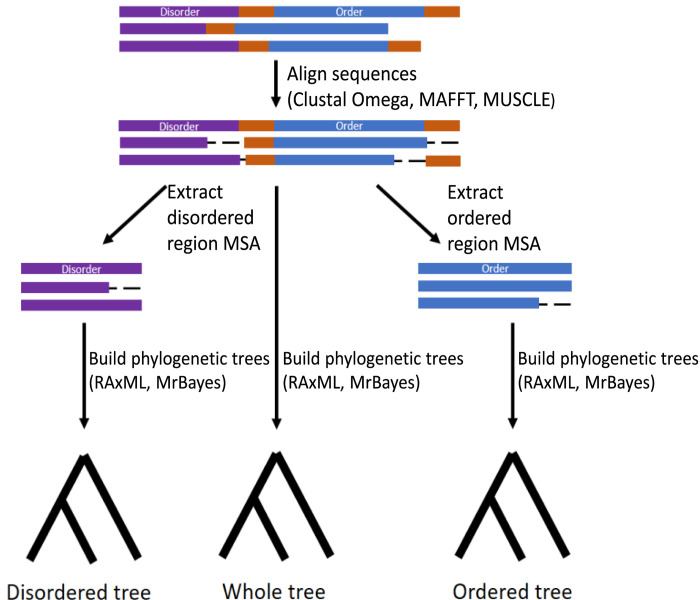
Process for generating whole, disordered, and ordered region MSAs and trees from mixed protein sequences. Purple regions represent disorder, blue regions represent order, orange regions represent areas of the sequence that were not identified as disordered or ordered regions, and dashed lines represent gaps.

Fig 1 represents the procedure for how MSAs and phylogenetic trees were obtained for the mixed proteins. MSAs were created for DNA sequences (without stop codons) and peptide sequences using Clustal Omega, MAFFT, and MUSCLE on the Emboss website [31–34]. All packages were run with their default settings. The peptide MSAs were used to create codon alignments from the nucleotide sequences using PAL2NAL [35]. To obtain the disordered region MSA, the position of the disordered region, based on the initial protein, was located in and cut from the MSA of the whole sequence. This was repeated with the ordered region to obtain the ordered region MSA. In cases where the portion of a sequence’s length in the alignment was <40% of the length of the initial sequence’s region, the sequence was removed and the remaining sequences were re-aligned. This reduces the chance of including sequences that were missing either ordered or disordered regions because of loss through evolution or misannotation. The same alignment methods were applied to the fully ordered and disordered proteins.

The AlignStat package in R was used to determine the similarity between MSAs of the same proteins from Clustal Omega, MAFFT, and MUSCLE alignments [36]. The MSA similarity was calculated as follows: one of the MSAs being compared is designated the reference MSA, and for each site in the reference MSA the site with the most matches in the other MSA is identified. A match between sites occurs when the same nucleotide or amino acid from the same sequence is present in both sites. The number of matches in a site is divided by the number sequences in the MSA, less the number of gap matches, to calculate the site similarity. The site similarities are then averaged to get the overall MSA similarity. Every similarity was measured twice, alternating which MSA was used as the reference MSA.

The only tool designed specifically for aligning disordered proteins that we are aware of is KMAD [8]. However, KMAD is intended to be used for insertion free alignments for comparing a sequence of interest to a whole alignment, rather than creating an alignment for phylogenetic analysis [8].

### Evolutionary rate comparison

To compare the evolutionary rate between the ordered and disordered regions in the mixed proteins, the difference between the identity of every pair of ordered regions and every pair of disordered regions was found using a similar method as described by Brown *et al*. [3]. Briefly, the pairwise identity for every sequence pair in both the disordered and ordered MSAs was calculated, then the difference between the pairwise identities was determined for every sequence pair. All the differences were averaged to obtain the average difference in identity between every protein’s ordered and disordered regions. This was done for all three peptide alignment methods (Clustal Omega [37], MAFFT [32] and MUSCLE [33]). In order to determine the significance of each difference, the ordered and disordered MSAs of each protein for each alignment method were scrambled before calculating the average difference in identity; this procedure was repeated 1000 times. The scrambled average differences in identity were used to create a probability distribution, and a two-sided test was performed to calculate the *p*-values for each pairwise identity difference.

### Phylogenetic trees

Phylogenetic trees were created from the MSAs by maximum likelihood using RAxML v8.2.12 [38] and by Bayesian inference using MrBayes 3.2.7a [39]. All trees constructed from the DNA and codon MSAs used a general time reversible substitution model with gamma-distributed rate variation. The PROTGAMMAAUTO option was used to automatically determine which models should be used for the peptide MSAs in RAxML. For trees built from peptide MSAs using MrBayes, fixed amino acid rate matrices were used. The peptide runs were performed without heated chains. For each RAxML run, 100 trees were created, and all the trees were rooted on the outgroup protein. The Adam consensus tree for these 100 trees was created by using the Bio.Phylo.Consensus module from Biopython [28,40,41]. All runs of MrBayes were performed for 5,000,000 generations, or until the average standard deviation of split frequencies was less than 0.01. A sampling frequency of 1000 was used and the consensus trees were created using a burn in fraction of 0.25.

#### Tree comparison

The Lee-Ashlock distances between trees (i.e. the distances between trees created from MSAs and the species tree) were calculated using the minimum containing clade method [42]. Briefly, for every pair of leaves in a tree, the minimum containing clade is the smallest clade that contains both leaves. If two trees have the same set of leaves, then the minimum containing clade can be compared between every pair of leaves by treating the minimum containing clade for every pair of leaves in a tree as a vector, and then computing the Euclidean distance between the vectors of each tree. The distance between trees was normalized by estimating the maximum distance between trees with an equivalent number of leaves. To create this estimate, one million pairs of random trees with the same number of taxa was generated, and the largest distance between pairs was selected as the maximum distance. Trees were also compared using the normalized Robinson-Foulds distance, which was calculated using the phangorn package (2.11.1) in R [43,44].

It is also important to note that all of the distances between MSA trees and the species tree are a measure of precision rather than accuracy. It cannot be guaranteed that the species tree used completely reflects how mammals actually evolved and accurately represents how each protein evolved. The species tree is acting as an approximation of the correct tree and a fixed point to compare other trees. Thus, distances to the species tree estimates an MSA tree’s precision, rather than its accuracy which would require a known correct tree.

## Results

### Mixed protein data collection

Analysis of the MSAs was first performed using mixed proteins (proteins with both disordered and ordered regions). A total of 14 initial mixed protein sequences were collected from the DisProt 8.0 database [21,22] and all of the proteins have at least one contiguous region of disorder >30 residues long, and at least one Gene 3D domain [23]. A summary of the proteins collected and several of their properties are shown in Table 1. The total number of sequences collected for each protein ranged from 86 to 116, and are represented by species throughout the Mammalia tree. The initial sequence came from *Homo sapiens*, with the exceptions of melanophilin and smoothelin-like protein 1, which came from *Mus musculus*. For the 14 sets of proteins, nine MSAs were created using Clustal Omega, MAFFT, and MUSCLE. Six MSAs were created using DNA and peptide sequences [31–33], while the remaining three MSAs were created by back translating the peptide alignments to obtain codon alignments. Once disordered and ordered regions MSAs were cut from the whole sequence MSAs, each protein had a total of 27 alignments: nine whole sequence MSAs, nine disordered region MSAs, and nine ordered region MSAs.

**Table 1:** List of mixed proteins. Summary of initial sequences collected, total number of sequences collected and their length properties. The lengths are based on the number of residues in the initial sequences.

Protein	DisProt ID	Whole Sequence Length	Ordered Region Length	Disordered Region Length	Number of Sequences Collected
**Anamorsin**	DP01137	312	172	93	116
**Beclin-1**	DP01149	450	210	150	103
**β-adducin**	DP00241	726	257	318	105
**DNA topoisomerase 1**	DP00075	765	429	203	93
**Galectin-3**	DP01332	250	135	112	100
**Histone H1**	DP00097	194	65	84	86
**Melanophilin**	DP00541	590	146	257	115
**p53**	DP00086	393	200	93	106
**Protein Tob1**	DP00794	345	115	223	90
**Proto-oncogene c-Fos**	DP00078	380	56	165	99
**Septin-4**	DP00537	478	296	119	87
**Smoothelin-like protein 1**	DP00742	459	107	341	114
**Telethonin**	DP00797	167	77	84	94
**Transcription factor p65**	DP00085	551	296	124	103

### Mixed protein MSA comparisons

In order to compare the evolutionary rates of the disordered and ordered regions, the difference between the averaged pairwise identity of the sequences in the disordered region and the ordered regions were calculated for each of the 14 proteins using the peptide MSAs (Table 2). These were then compared to the differences between scrambled MSAs of the disordered and ordered region of the same protein to assess if the observed differences were more significant than expected by random chance. Of the 14 proteins, 12 were found to have significantly higher identities in their ordered region (*p*-values ≪ 0.05). The anamorsin Clustal Omega and MAFFT MSAs and all telethonin MSAs were not found to have a significant difference in the identity between their ordered and disordered regions. Only the anamorsin MUSCLE MSA had a disordered region with a significantly higher identity than the ordered region.

**Table 2:** Average pairwise identity difference between disordered and ordered regions. Δ_identity_ is the difference between the average pairwise identity of the disordered region and the average pairwise identity of the ordered region. *p*-values were considered significant at <0.05.

Proteins	Clustal Omega		MAFFT		MUSCLE	
	Δ_identity_	*p*-value	Δ_identity_	*p*-value	Δ_identity_	*p*-value
**Anamorsin**	0.054	0.070	0.039	0.154	0.060	0.047
**Beclin-1**	-0.116	0.001	-0.072	0.001	-0.069	0.001
**β-adducin**	-0.070	0.001	-0.051	0.001	-0.082	0.001
**DNA topoisomerase 1**	-0.106	0.001	-0.134	0.001	-0.164	0.001
**Galectin-3**	-0.156	0.001	-0.266	0.001	-0.142	0.001
**Histone H1.0**	-0.168	0.001	-0.074	0.001	-0.132	0.001
**Melanophilin**	-0.403	0.001	-0.456	0.001	-0.371	0.001
**p53**	-0.409	0.001	-0.338	0.001	-0.406	0.001
**Protein Tob1**	-0.154	0.001	-0.149	0.001	-0.069	0.001
**Proto-oncogene c-Fos**	-0.038	0.001	-0.107	0.001	-0.116	0.001
**Septin-4**	-0.286	0.001	-0.204	0.001	-0.271	0.001
**Smoothelin-like protein 1**	-0.468	0.001	-0.509	0.001	-0.331	0.001
**Telethonin**	-0.016	0.524	-0.049	0.134	-0.047	0.140
**Transcription factor p65**	-0.097	0.001	-0.081	0.001	-0.139	0.001

To determine how consistent the disordered and ordered regions alignments were between the three methods, the similarities between the MSAs were calculated as described in the Materials and Methods. To ensure that the comparisons between MSAs are valid, identical sequences and regions of sequences must be compared. Therefore, whole sequence MSAs were initially used, and the sites that aligned with the disordered region and the ordered region from the initial sequence were identified in the reference MSA. The similarities between these sites were used to estimate the similarity between the disordered region MSAs and between the ordered region MSAs. For each pairwise MSA comparison, the similarity was measured twice to alternate which MSA was used as the reference MSA in order to remove bias from using only one of the MSAs as the query. These similarity values were then averaged. The similarity between every combination of Clustal Omega, MAFFT, and MUSCLE MSA using disordered and ordered regions was computed for a total of six comparisons (S1 and S2 Tables). These similarities were averaged based on sequence type and regions of order and disorder, and a summary of these results can be found in Table 3; codon MSA similarities were not included since they are identical to the peptide similarities. As can be seen in Table 3, the averaged similarity values for the ordered region MSAs were higher than the disordered region MSAs with the exceptions of anamorsin and telethonin. Twelve proteins had ordered region MSAs that had a similarity of 0.95 or greater, with the lowest value being 0.88 for the peptide MSAs of melanophilin. By comparison, only eight proteins had disordered region MSAs that had a similarity of 0.90 or greater.

**Table 3:** The average similarity between different MSAs of disordered and ordered regions. The similarity was calculated using AlignStat [36]. The pairwise identities were averaged from the Clustal Omega MSA, the MAFFT MSA, and the MUSCLE MSA.

Proteins	Disordered Region		Ordered Region	
	DNA	Peptide	DNA	Peptide
**Anamorsin**	0.993	0.993	0.948	0.964
**Beclin-1**	0.904	0.946	0.968	0.973
**β-adducin**	0.977	0.966	1.000	1.000
**DNA topoisomerase 1**	0.665	0.663	0.999	0.998
**Galectin-3**	0.448	0.530	0.987	0.989
**Histone H1.0**	0.955	0.960	1.000	1.000
**Melanophilin**	0.481	0.636	0.914	0.880
**p53**	0.664	0.658	0.994	0.986
**Protein Tob1**	0.810	0.813	1.000	1.000
**Proto-oncogene c-Fos**	0.946	0.954	0.999	1.000
**Septin-4**	0.915	0.915	1.000	1.000
**Smoothelin-like protein 1**	0.544	0.643	0.927	0.999
**Telethonin**	0.967	0.980	0.918	0.977
**Transcription factor p65**	0.924	0.946	0.983	0.986

### Using normalized Lee-Ashlock distance to compare trees

**Fig 2:**
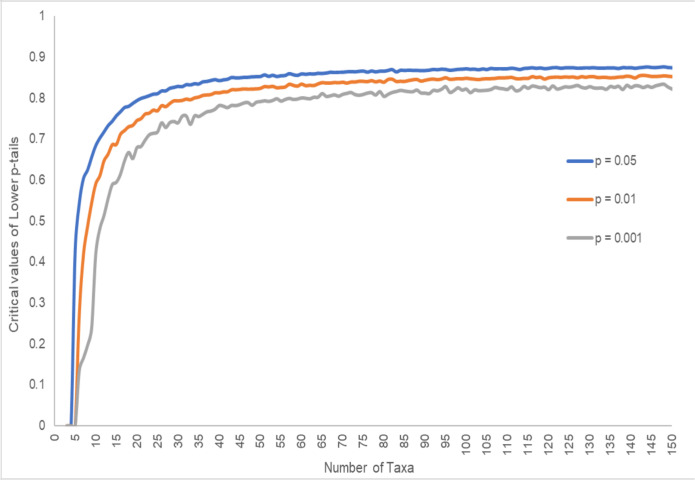
Critical values for the lower p-tail of distances between 10,000 pairs of random trees for different number of taxa. The normalized Lee-Ashlock distance was used to calculate all distances. Blue line, p = 0.05; orange line, p = 0.01; grey line, p = 0.001.

Our next goal was to compare phylogenetic trees inferred from MSAs to the species tree. Such an approach requires a method of comparing trees, such as the Lee-Ashlock distance. While it is possible to compare the Lee-Ashlock distances between trees with the same of number of taxa, with an increasing number of taxa there is an increase in the maximum possible Lee-Ashlock distance between trees. Additionally, the distribution of tree distances changes with the number of taxa. In order to compare proteins with different numbers of taxa such as that in our analyses, the Lee-Ashlock distances were normalized using the estimated largest distance between two trees for a given number of taxa. To determine at what normalized distance two trees were significantly close, the distance between 10,000 pairs of random trees was calculated. This was done for trees with 3 to 150 taxa, since trees with less than 3 leaves are identical and 150 exceeds the number of sequences used (86 to 116 taxa). These distances were then treated as a probability distribution, and the critical values of the lower p-tails were obtained for *p* = 0.05, *p* = 0.01, and *p* = 0.001 (Fig 2). Critical values rapidly increased until ~20 taxa, and started to plateau at ~60 taxa. For 86 and 116 taxa, the critical values for *p* = 0.05, 0.01, and 0.001 ranged from 0.867 to 0.874, 0.841 to 0.852, and 0.811 to 0.830, respectively.

**Fig 3:**
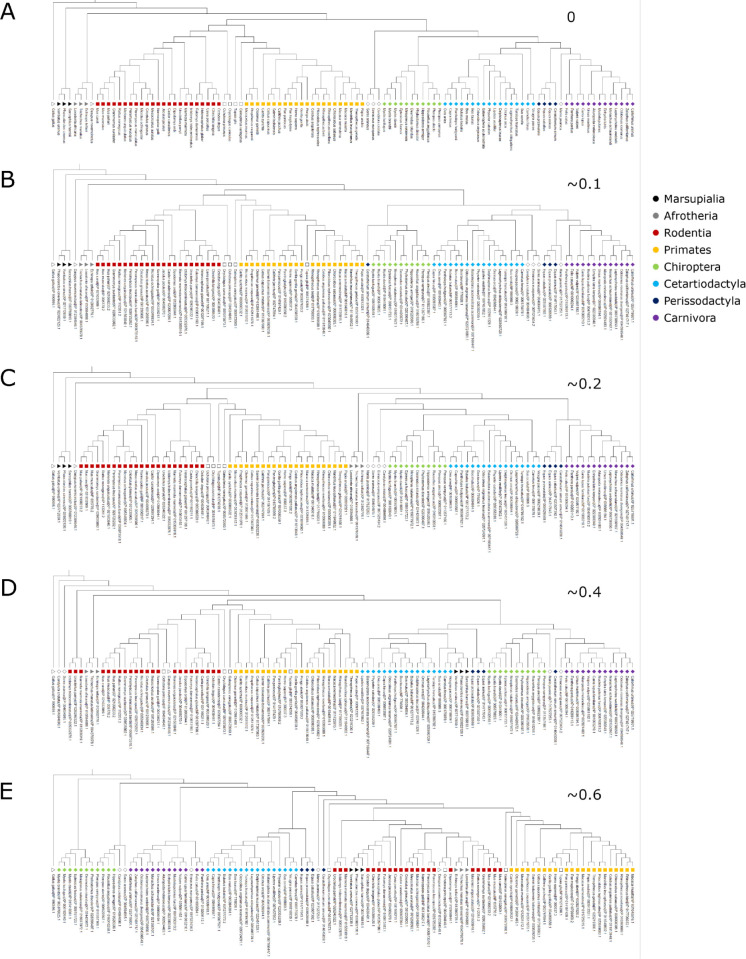
Phylogenetic trees at different distances from the species tree. Squares represent members of Eurachontoglires, diamonds represent members of Laurasiatherian, and triangles represent all other taxa. The symbols cluster the taxa on orders and superorders; the color legend is shown in the figure. Symbols with no colour fill represent all other taxa. Tree A) is the species tree from TimeTree [19] and therefore has a distance of zero. Each tree (B-E) represents an approximate distance to the species tree, where the precise distance for each tree was: B) 0.092 for ~0.1, C) 0.198 for ~0.2, D) 0.381 for ~0.4, and E) 0.600 for ~0.6.

To gain an intuitive understanding of how the normalized Lee-Ashlock distances relate to differences between the trees, Fig 3 shows tree 0 (the species tree), and trees that are ~0.1, ~0.2, ~0.4, and ~0.6 normalized Lee-Ashlock units away from the species tree. These trees were collected from the sets of RAxML trees used to create the p53 consensus trees using RAxML. p53 was selected because its trees covered a broad range of distances depending on the MSA method and part of the p53 sequence used (whole, ordered and disordered region), and by selecting one protein set all of these trees have the exact same species present so they can be readily compared. These trees are not meant to be a “best attempt”, but instead represent a sampling to provide a range of normalized Lee-Ashlock distances. In Fig 3, nearly all taxa are placed into one of eight different groups: Marsupialia, Afrotheria, Rodentia, Primates, Chiroptera, Cetartiodactyla, Perissodactyla, and Carnivora. These groupings are based on orders and superorders from TimeTree, with the exception of Marsupialia, which is an infraclass [19].

Generating these trees with different distances from the species tree gives us examples of what types of shifts may occur as a phylogenetic tree becomes less similar to a species tree. For Fig 3B, representing a tree that is relatively close to the species tree (Fig 3A), some of the most noticeable changes involve a singular taxon that is out of place. One taxon that is not a member of the Primates group falls into the Primates clade; however, this taxon (*Tupaia glis*) is a species closely related to primates, and falls just outside the Primates clade in the species tree. Another deviation of Fig 3B from the species tree occurs with *Ceratotherium simum*, a member of the Perissodactyla group, which is placed next to the Chiroptera clade away from any other members of the Perissodactyla group. The smallest clade that would include all members of the Perissodactyla group would also include all of Chiroptera, Cetartiodactyla, and Carnivora. All other groups can be placed into clades that just contain members of their group in Fig 3B, and the evolutionary history (connections between clades) of the clades matches the evolutionary history of the same clades in the species tree. In the case of Fig 3C (distance ~0.2), all of the taxa that are members of a group clade can be placed into a clade that contains every member of their group with no other taxa. However, the connections between clades do not match the species tree as well when compared to Fig 3B; Afrotheria is not placed close to the root of the tree, and is instead placed in between Eurachontoglires (Rodentia and Primates) and Laurasiatheria (Chiroptera, Cetartiodactyla, Perissodactyla, and Carnivora). Additionally, Cetartiodactyla first fall into a clade with Chiroptera, while in the species tree the Cetartiodactyla is initially placed into a clade with Carnivora and Perissodactyla.

For Fig 3D (distance ~0.4), the only group clades that contained only members of their group are Chiroptera, Afrotheria, and Marsupialia. Additionally, the connections between groups differ from the species tree. Carnivora are placed in a clade with Chiroptera before Cetartiodactyla, and Marsupialia are placed in a clade that contains all of the Cetartiodactyla members, rather than near the root of the tree. There are also three members of Rodentia that fell near the root of the tree, which is away from the rest of the Rodentia group. Fig 3E (distance ~0.6) also had three group clades that contained just members of their group: Cetartiodactyla, Afrotheria, and Marsupialia. However, all of the groups that are members of Laurasiatheria are placed near the root of the tree. This is due to the outgroup (*G*. *gallus*) being placed near the Chiroptera clade, while in all other trees it is placed closer to split between Laurasiatheria and Eurachontoglires.

The presence of the Laurasiatheria and Eurachontoglires split in Fig 3E highlights a potential problem with using the minimum containing clade method when calculating the Lee-Ashlock distance. This split still exists in the Fig 3E if the tree is unrooted. However, in the rooted tree, all of the Eurachontoglires are placed within the Laurasiatheria clade. These results show that changing the root of the tree could greatly alter the distance to the species tree. If Fig 3E and the species tree were instead rooted on *Homo sapiens*, the distance between the trees decreases from 0.600 to 0.304. This indicates that if the chosen outgroup is far from its expected location, it can greatly alter the topology of the tree and increase the distance between trees. In order to reduce the bias of rooting on a single leaf, the distance between the trees of interest and the species tree was calculated for every leaf at the root of the tree. Instead of a single distance, a set of distances equal to the number of leaves in the trees was obtained. Using this new method, the averaged distances for each tree in Fig 3B–3E are 0.167, 0.219, 0.349, and 0.387 respectively. All trees are still in the same order in distance from the species tree, however, comparing all possible rooted trees changes the magnitude of the distances to some degree. Using the normalized Robinson-Foulds distance, the distances between the trees are 0.175, 0.165. 0.524, and 0.515 for Fig 3B–3E respectively. These distances better match the Lee-Ashlock distances calculated using every root, with the two closest trees and the two furthest trees being more similar distances to the species tree than initially calculated. However, the proximity to the species tree was swapped for the two closest and furthest trees.

### Mixed protein phylogenetic tree precision

In order to measure the tree precision of the inferred trees to the species tree, the MSAs of the 14 proteins were used to create several phylogenetic trees. Trees were created with RAxML [38] and MrBayes [39] using DNA, codon, and peptide alignments to check if results were consistent across multiple tree building approaches. In total, 54 consensus trees were created for each protein using the two tree building methods and the sets of 27 MSAs. The normalized Lee-Ashlock distances between these trees and the species tree were determined and used to compare the precision of the phylogenetic trees in order to evaluate the MSAs, where precision is defined as the phylogenetic tree’s proximity to the species tree.

Initial comparisons were made between trees from the disordered and ordered regions MSA from the same mixed protein and the same alignment (same sequence type and alignment method). The Wilcoxon two-sided test was used to determine if the set of distances from the disordered tree were significantly different (*p*-value < 0.05) from the set of distances from the ordered tree, and the distance sets were averaged to determine which was closer to the species tree. Table 4 summarizes how frequently either the disordered consensus tree, ordered consensus tree, or neither were significantly closer to the species tree. The frequencies for the RAxML and MrBayes trees were treated separately and together as a combined frequency to provide an overall ranking. Based on the combined frequencies, eight of the 14 proteins had disordered trees that were more frequently closer to the species tree, and four of the 14 proteins had ordered trees that were more frequently closer to the species tree. However, of the eight proteins (proto-oncogene c-Fos, smoothelin-like protein 1, septin-4, histone H1.0, β-adducin, protein Tob1, melanophilin, and beclin-1), melanophilin and beclin-1 had a disagreement between the RAxML and the MrBayes consensus trees as to whether ordered or disordered trees were closer to the species tree. For melanophilin, the disordered RAxML trees were more frequently found closer to the species tree, while for beclin-1, the disordered MrBayes trees were more frequently found closer to the species tree.

**Table 4:** Frequency in which disordered and ordered consensus trees were significantly closer to the species tree. The frequencies were calculated separately and were also combined for the RAxML and MrBayes trees. A tree was considered closer if they were significantly different from the opposite region tree for the same MSA method (*p*-value < 0.05 using Wilcoxon two-sided test).

Proteins	Combined			RAxML			MrBayes		
	Disorder closer	Order closer	Neither closer	Disorder closer	Order closer	Neither closer	Disorder closer	Order closer	Neither closer
**Proto-oncogene c-Fos**	1.00	0.00	0.00	1.00	0.00	0.00	1.00	0.00	0.00
**Smoothelin-like protein 1**	1.00	0.00	0.00	1.00	0.00	0.00	1.00	0.00	0.00
**Septin-4**	0.94	0.00	0.06	0.89	0.00	0.11	1.00	0.00	0.00
**Histone H1**	0.94	0.06	0.00	1.00	0.00	0.00	0.89	0.11	0.00
**β-adducin**	0.89	0.11	0.00	0.89	0.11	0.00	0.89	0.11	0.00
**Protein Tob1**	0.78	0.17	0.06	0.89	0.00	0.11	0.67	0.33	0.00
**Melanophilin**	0.61	0.33	0.06	0.89	0.11	0.00	0.33	0.56	0.11
**Beclin-1**	0.44	0.39	0.17	0.33	0.67	0.00	0.56	0.11	0.33
**Telethonin**	0.33	0.22	0.44	0.56	0.11	0.33	0.11	0.33	0.56
**Galectin-3**	0.44	0.44	0.11	0.33	0.67	0.00	0.56	0.22	0.22
**DNA topoisomerase 1**	0.28	0.50	0.22	0.33	0.22	0.44	0.22	0.78	0.00
**Transcription factor p65**	0.33	0.67	0.00	0.33	0.67	0.00	0.33	0.67	0.00
**p53**	0.06	0.89	0.06	0.11	0.89	0.00	0.00	0.89	0.11
**Anamorsin**	0.06	0.94	0.00	0.11	0.89	0.00	0.00	1.00	0.00

Four of the 14 proteins (anamorsin, p53, transcription factor p65, and DNA topoisomerase 1) had ordered trees that were more frequently closer to the species tree. Among these proteins, only DNA topoisomerase 1 had disagreement between the RAxML and the MrBayes consensus trees, with the ordered MrBayes trees was more frequently closer to the species tree. Galectin-3 had ordered and disordered trees that were closer to the species tree an equivalent number of times, and telethonin had disordered and ordered tree distances to the species tree that were most frequently not significantly different.

**Fig 4:**
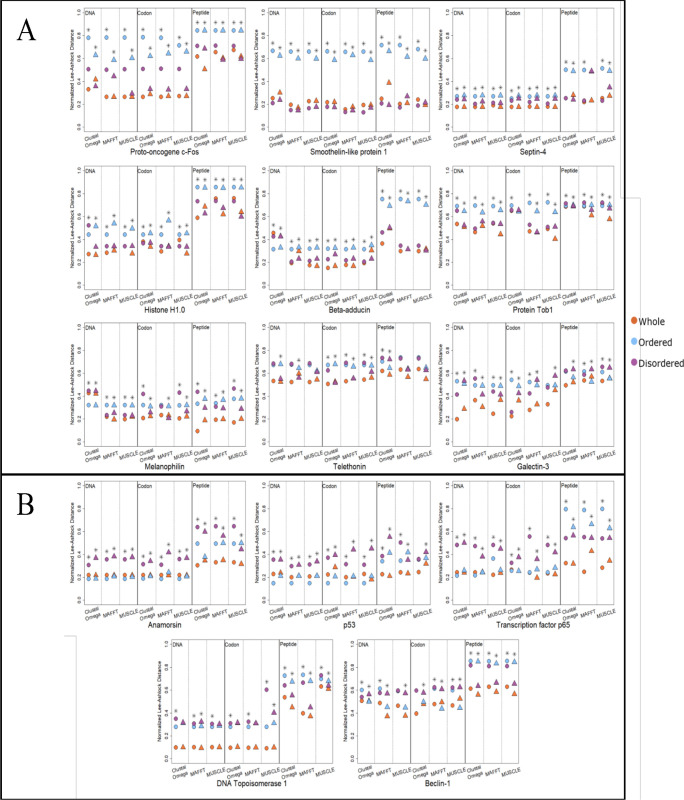
Distance of MrBayes and RAxML consensus trees to the species tree from alignments of whole, ordered region, and disordered region sequences. (A) Proteins where the disordered consensus tree is closer to the species tree compared to the ordered consensus tree. (B) Proteins where the ordered consensus tree is closer to the species tree than the disordered consensus tree. Circles represent averaged MrBayes consensus tree distances and triangles represent averaged RAxML consensus tree distances. Orange, whole protein sequence; blue, ordered region sequence; purple, disordered region sequence. *indicates where disordered region trees and ordered region trees were significantly different to each other (*p*-value < 0.05 using Wilcoxon two-sided test).

The precision of the best disordered consensus trees was compared to the precision of the best ordered consensus trees to check if there was consistency between the best overall trees and how frequently the trees did better. The best tree was determined by averaging all the distances for each consensus tree, and then selecting the tree that was closest to the species tree. The results are divided into two groups: proteins where the best disordered tree was closer to the species tree than the best ordered tree (Fig 4A), and proteins where the best ordered tree was closer to the species tree than the best disordered tree (Fig 4B). The proteins with the best disordered trees were proto-oncogene c-Fos, smoothelin-like protein 1, septin-4, histone H1.0, β-adducin, protein Tob1, melanophilin, and galectin-3. The proteins with the best ordered trees were anamorsin, p53, transcription factor p65, DNA topoisomerase 1, and beclin-1. The region with the best overall trees was also the region that produced trees that were more frequently closer to the species tree (based on the combined frequency from Table 4), with the two exceptions of galectin-3 and beclin-1.

In Fig 4, the whole sequence trees were also included, since it would be expected that these trees would be closer to the species tree than the disordered or ordered trees because they came from MSAs that should contain the most information (i.e., from both disordered and ordered regions), along with other sequence that was not part of either region. In these results, the majority of the proteins had at least one whole sequence consensus tree that was closer to the species tree than 0.2. Anamorsin, histone H1.0 and protooncogene c-Fos all had at least one whole sequence consensus tree that was closer to the species tree than 0.3, while every whole sequence consensus tree for beclin-1, protein Tob1, and telethonin that was further than 0.3 from the species tree. However, there were several cases where the disordered or ordered region MSAs produced phylogenetic trees closer to the species tree than those from whole sequence MSAs. Anamorsin and p53 had ordered region trees that were consistently better than the whole sequence trees, while the only protein with disordered region trees that was consistently better than the whole sequence trees was smoothelin-like protein 1.

Fig 4 and Table 4 compared the disordered region to the ordered region from the same protein. To check if one region overall had trees that were significantly closer to the species tree, all of the disordered region trees and all of the ordered region trees were compared across all of the proteins, where comparisons were made between the disordered and ordered region consensus trees from MSAs with the same method and sequence type (S3 Table). The disordered and ordered region consensus trees were not found to be significantly different (*p*-value < 0.05 using Wilcoxon two-sided test), with the exception of RAxML trees from MAFFT and MUSCLE peptide alignments, where the disordered consensus trees were closer to the species tree for both.

However, when comparisons were made between the ordered trees from the same MSA method but different sequence types, it was found that all comparisons of codon or DNA to peptide were significantly different (S3 Table); i.e., the codon and DNA trees were closer to the species tree than the peptide trees. The same comparisons were made for the whole and disordered trees; out of six comparisons between codon or DNA trees to peptide trees, four were found to be significantly different for both whole and disordered trees, with the codon and DNA trees being found closer to the species tree (S3 Table). Comparisons between DNA and codon trees were never found to be significantly different.

The normalized Robinson-Foulds distance was also used to compare the disordered and ordered region trees of the mixed proteins (S1 Fig). In general, a similar pattern of disordered region trees being closer to the species tree was observed. However, galectin-3 and telethonin more consistently had ordered region trees closer to the species tree, while transcription factor p65 more consistently had disordered region trees closer to the species tree.

### Mixed protein tree precision and MSA correlation

**Fig 5:**
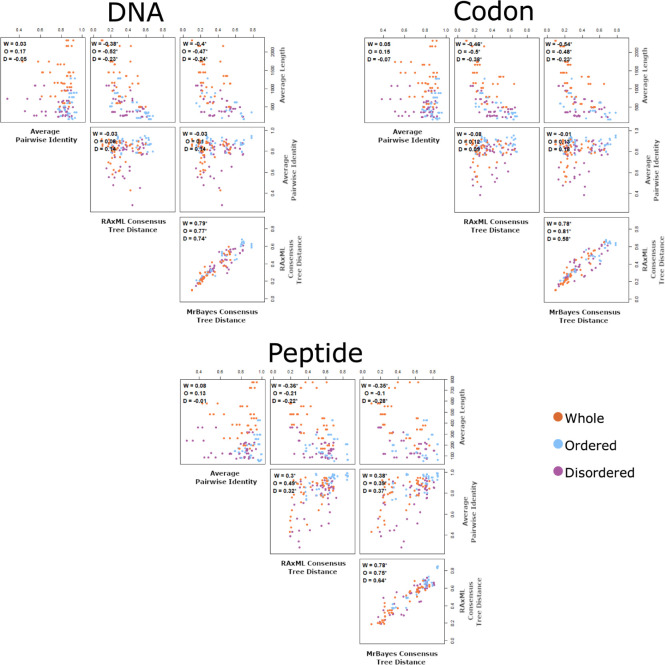
Correlations between multiple sequence alignment features and distances of the consensus trees to the species tree of the mixed proteins. The Kendall rank correlation was calculated for whole sequence MSAs (W), ordered region MSAs (O), and disordered region MSAs (D), and are shown as an inset in the upper left corner. Orange, whole protein sequence; blue, ordered region sequence; purple, disordered region sequence. The correlations for whole sequence MSAs, ordered region MSAs, and disordered region MSAs were calculated separately. *indicates correlation that were significant (*p*-value < 0.05).

Other features of MSAs were compared to determine in what way they may affect how well the species tree was recaptured. The Kendall rank correlations, a measure of dependence between two variables, was determined between the average length of the sequences, the average pairwise identity, the RAxML consensus tree distances, and the MrBayes consensus tree distance (Fig 5). The strongest correlations occurred when tree distances from RAxML were compared to tree distances from MrBayes. The next strongest correlations were between the sequence lengths and tree distances, which was found to be consistently negative, showing that longer sequences had trees closer to the species tree. For the DNA and codon MSAs, the whole sequences and ordered regions had stronger correlations than the disordered regions. However, in the case of the peptide MSAs, the ordered regions did not have a correlation that was significantly different than zero (*p*-values > 0.05). Both whole sequence and disordered region correlations were significant, with the whole sequences having a stronger correlation. In the case of correlations between the average pairwise identity and the tree distances, DNA and codon MSAs were not found to be significantly different from zero. All of the correlations were significant for the peptide MSAs.

### Fully disordered and fully ordered protein data collection

To determine the robustness of the patterns observed with the mixed sequences, comparisons of the MSAs from fully disordered proteins and fully ordered proteins were also made. Nine fully disordered proteins were collected from the DisProt database, and a summary of these proteins is shown in Table 5. The initial sequence for each protein was collected from either *Homo sapiens*, *Bos taurus*, *Mus musculus*, or *Rattus norvegicus*. In order to match the same number of fully disordered proteins, the same number of fully ordered proteins were collected (Table 6). Sequences were collected from the UniProtKB using well-studied proteins with known structures and absent from the DisProt database. The initial sequence for all of these proteins came from *Homo sapiens*.

**Table 5:** Summary of fully disordered proteins collected. DisProt IDs are for the initial protein sequences that were collected.

Proteins	DisProt ID	Average length	Sequences collected
**β-casein**	DP00329	224.3	115
**Bone sialoprotein 2**	DP00332	296.9	131
**Chromogranin-A**	DP00118	433.0	130
**Ermin**	DP01521	281.4	130
**Involucrin**	DP00221	399.6	115
**Osteopontin**	DP00214	290.7	120
**Protein LBH**	DP00661	111.8	106
**Prothymosin α**	DP00814	116.8	71
**Securin**	DP00521	200.6	123

**Table 6:** Summary of fully ordered proteins collected. UniProtKB IDs are for the initial protein sequences that were collected.

Proteins	UniProtKB ID	Average length	Sequences collected
**Alcohol dehydrogenase class-3**	P11766	377.4	120
**β-galactosidase**	P16278	639.8	114
**Carbonic anhydrase 1**	P00915	260.4	99
**Citrate synthase**	O75390	465.1	129
**Hemoglobin subunit α**	P69905	141.2	115
**Hexokinase-1**	P19367	911.5	123
**Insulin**	P01308	88.3	117
**L-lactate dehydrogenase A chain**	P00338	333.1	92
**Succinate CoA ligase [ADP forming] subunit β**	Q9P2R7	459.8	145

### Fully disordered and fully ordered protein MSA comparisons

A total of nine of MSAs were created for each fully disordered and ordered protein. Similar to the mixed proteins, an MSA was created for every combination of sequence type (DNA, codon, and peptide) and MSA method (Clustal Omega, MAFFT, and MUSCLE). The similarity between these DNA and peptide MSAs were found for both the fully disordered proteins and the fully ordered proteins and the average pairwise identity was calculated for the DNA, codon, and peptide alignments (Table 7). In the case of the disordered MSAs, only two of the nine proteins had MSAs with similarity scores higher than 0.90, and only securin had an average pairwise identity >0.75. The ordered MSAs had much higher similarity and average pairwise identity than the disordered MSAs, where all of the similarity scores were >0.90 and all of the pairwise identities were >0.75. The only exception was insulin, which had a peptide MSA similarity of 0.242 and a DNA MSA similarity of 0.833. The identities for insulin were 0.663, 0.707, and 0.625 for DNA, codon, and peptide MSAs, respectively.

**Table 7:** The average similarity between different MSAs of fully disordered and fully ordered proteins. The similarity was calculated using AlignStat [36]. The pairwise identities were averaged from the Clustal Omega MSA, the MAFFT MSA, and the MUSCLE MSA.

	MSA Similarity			Average Pairwise Identity		
Protein	Type	DNA	Peptide	DNA	Codon	Peptide
**β-casein**	Disorder	0.648	0.448	0.587	0.561	0.449
**Bone sialoprotein 2**	Disorder	0.850	0.879	0.728	0.729	0.679
**Chromogranin-A**	Disorder	0.711	0.790	0.652	0.671	0.574
**Ermin**	Disorder	0.899	0.914	0.732	0.719	0.636
**Involucrin**	Disorder	0.277	0.316	0.391	0.384	0.278
**Osteopontin**	Disorder	0.740	0.470	0.690	0.686	0.588
**Protein LBH**	Disorder	0.788	0.788	0.722	0.729	0.733
**Prothymosin α**	Disorder	0.738	0.771	0.713	0.712	0.748
**Securin**	Disorder	0.866	0.934	0.793	0.777	0.742
**Alcohol dehydrogenase class-3**	Order	0.973	0.976	0.855	0.844	0.899
**β-galactosidase**	Order	0.953	0.955	0.765	0.762	0.753
**Carbonic anhydrase 1**	Order	0.991	0.993	0.800	0.787	0.763
**Citrate synthase**	Order	0.973	0.982	0.860	0.866	0.915
**Hemoglobin**	Order	1.000	1.000	0.817	0.818	0.788
**Hexokinase-1 subunit α**	Order	0.961	0.967	0.855	0.858	0.902
**Insulin**	Order	0.833	0.242	0.663	0.707	0.625
**L-lactate dehydrogenase A chain**	Order	0.996	0.997	0.879	0.870	0.924
**Succinate CoA ligase [ADP forming] subunit β**	Order	0.914	0.942	0.868	0.861	0.887

#### Fully disordered and ordered protein phylogenetic tree precision

**Fig 6:**
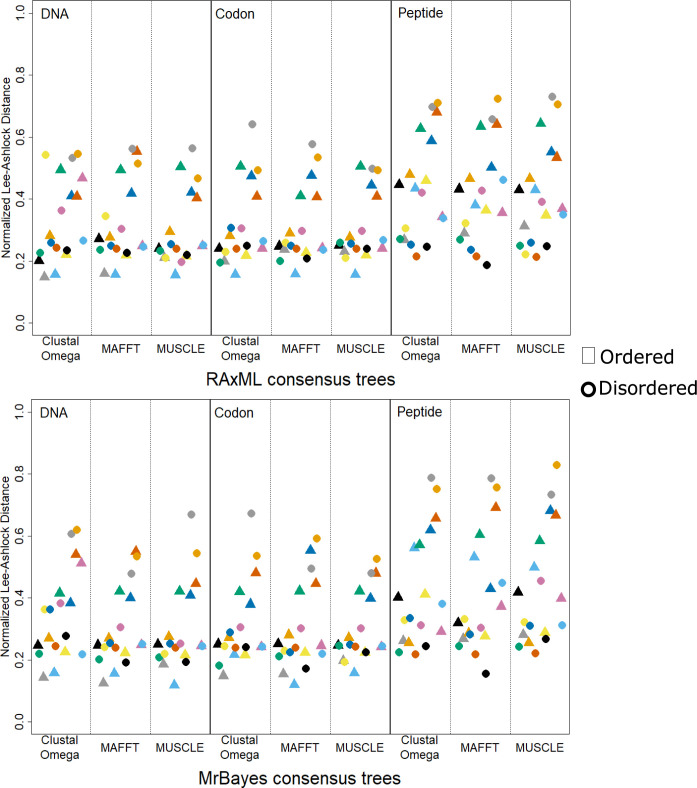
Distance of MrBayes and RAxML consensus trees to the species tree for fully disordered and ordered proteins. Ordered proteins (represented by triangles): ▲ alcohol dehydrogenase; ▲ β-galactosidase; ▲ carbonic anhydrase 1; ▲ citrate synthase; ▲ hemoglobin; ▲ hexokinase-1; ▲ insulin; ▲ LDHA; ▲ succinate CoA ligase (ADP forming). Disordered proteins (represented by circles): ● β-casein; ● bone sialoprotein; ● chromogranin-A; ● ermin; ● involucrin; **●** osteopontin; ● protein LBH; ● prothymosin α; ● securin.

The MSAs of the fully disordered and ordered proteins were used to create phylogenetic trees, which were built and compared to the species tree using the same methods as those used for the mixed proteins. The distances to the species tree were similarly calculated in order to compare the precision of the fully disordered trees to the precision of the fully ordered trees. However, unlike the mixed proteins, where comparisons could be made between ordered and disordered regions within one protein, only comparisons across all of the fully ordered and disordered proteins could be made. Comparisons between the fully disordered and ordered tree distances were made for every combination of sequence type and MSA method (Fig 6). In all comparisons, none of the fully disordered and ordered tree distances were significantly different from one another (*p*-value > 0.05 using the Wilcoxon two-sided test). Comparisons of fully disordered and ordered trees was also performed using the normalized Robinson-Foulds distance (S2 Fig). Likewise, none of the fully disordered and ordered tree distances were significantly different (*p*-value > 0.05 using the Wilcoxon two-sided test).

#### Fully disordered and ordered protein tree precision and MSA correlation

**Fig 7:**
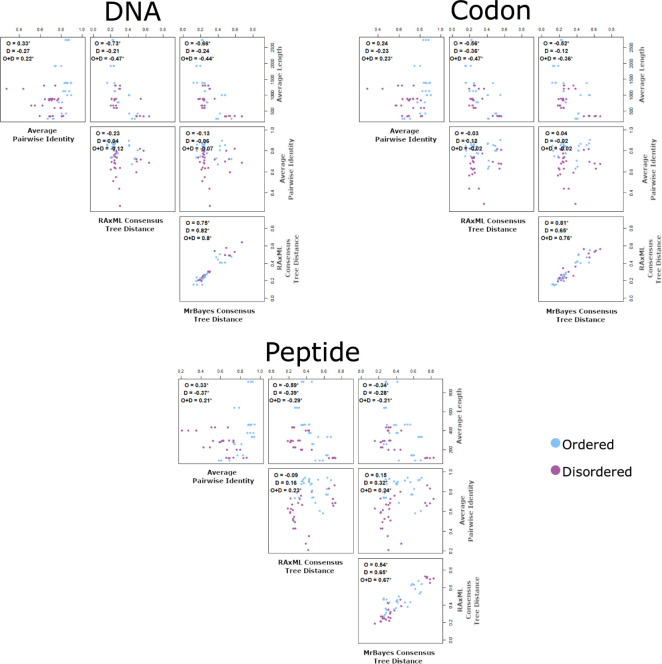
Correlation between multiple sequence alignment features and distances of the consensus trees to the species tree of fully ordered and disordered proteins. The Kendall rank correlation was calculated for the fully ordered proteins (O), the fully disordered proteins (D), and the combined (O+D). Blue, ordered sequence region; purple, disordered sequence region. *indicates correlations that were significant (*p*-value < 0.05).

Features from the fully disordered and fully ordered protein MSAs were compared (Fig 7), similar to the comparison made in Fig 5 for the mixed proteins. The disordered and ordered proteins were both treated separately and as a combination of both data sets. Analogous to what was observed with the mixed proteins, the strongest correlations occurred between the RAxML tree distances and the MrBayes tree distances, and the correlation was significant (p < 0.05) for every type of comparison. For all fully ordered protein MSAs, all average sequence length and tree distances were significantly negatively correlated (i.e., the longer the sequence the shorter the tree distance). These correlations were stronger than the correlations of the average sequence length and tree distances for the fully disordered proteins and the combined fully disordered and ordered proteins. The only times the average sequence length and tree distances were significantly correlated for the disordered proteins was for the peptide MSAs with trees created using RAxML and MrBayes, and the codon MSAs with trees created using RAxML. The only correlations where the averaged pairwise identity between sequences and tree distances were significantly correlated occurred in the fully disordered sequence sets and the combined sequence sets when using peptide MSAs.

## Discussion

### Assessment of MSAs

The study of IDPs is gaining increased attention, but we need to ensure that the tools we use are appropriate. As an example, MSAs are important tools in studying the function and evolutionary relationships of proteins. However, it has been suggested that disordered proteins are difficult to align because they rapidly evolve and experience more frequent indels [6–9]. Our goal was to compare and assess the quality of MSAs created from disordered sequences to these from ordered sequences using several different tools and datasets.

MSAs were initially assessed based on the similarity between the different MSAs [14–16]. The higher similarity scores of the ordered MSAs (Tables 3 and 7) could be considered an indicator that they are more correct, since if a correct MSA was found, it would be more likely to be found between different alignment methods [16].

There were only three exceptions to this observation. Anamorsin and telethonin had disordered region MSAs that were more similar than the ordered region MSAs. These proteins were also the only mixed proteins that had identities that were not significantly lower in their disordered regions compared to their ordered regions. Likewise, the only fully ordered protein with MSA similarity scores lower than those observed in the MSAs of the fully disordered proteins was insulin, which also had the lowest identity of all of the fully ordered proteins. This suggests that lower conservation in these sequences allows for greater variation in the MSAs produced, thus lower similarities between MSAs.

Additionally, the MSAs of disordered and ordered sequences were compared using the precision of the phylogenetic trees created from their MSAs, where precision was based on how close the inferred MSA tree was to the species tree. The comparisons between the disordered regions and ordered regions from the mixed proteins found, surprisingly, that the majority of them had disordered region MSAs that produced more precise trees. Of the 14 mixed proteins, 8 proteins had disordered regions that produced more precise phylogenetic trees for both RAxML and MrBayes trees (Table 4). This observation held even when comparing the best overall tree from the disordered region to the best overall tree from the ordered region (Fig 4). The ordered region produced better trees in only four of the mixed proteins, with the two remaining proteins being ambiguous as to whether the MSAs from the disordered or ordered regions produced more precise trees.

The initial comparisons of similarity between the MSAs does agree with the conventional yet unverified thought that disordered sequences are harder to align [6–9]. The lower similarities for the disordered proteins seem to be linked with lower pairwise identity, and one of the main reasons disordered proteins are believed to be difficult to align is the lower conservation between sequences [6]. However, comparisons of phylogenetic trees from disordered sequences and ordered sequences found that both were equally good at recapitulating the species tree. Although disordered and ordered sequences generating equally good trees may seem contradictory, it is important to consider the relationship between MSA precision and tree precision. Our method can only establish if an MSA is sufficiently accurate for phylogenetic tree reconstruction. Most importantly, if an MSA does poorly it does not confirm that the MSA is inaccurate or of low quality [16]. For example, an MSA of identical sequences would have all sites completely conserved and would be assumed to be correct for these sequences. However, these fully conserved sites would provide no information about the relatedness of the sequences and could not be used to build a phylogenetic tree. We cannot say the disordered MSAs were as good as the ordered MSAs because we cannot independently verify their precision with aligned structures. We can say that the MSAs from disordered sequences were as good at recapturing the species tree as the MSAs of the ordered sequences based on the data used.

Dessimoz and Gil similarly observed that alignment variability was a poor predictor of tree precision for peptide sequences [17]. Alignment variability was a better predictor of tree precision for DNA sequences, however other methods were found to be better [17]. Interestingly, Wang *et al*. observed with simulated data that the correlation between MSA precision and tree precision varied between sequences, and becomes stronger as sequences become more divergent [10]. Since IDPs tend to be less conserved, this could suggest that although disordered sequence sets and ordered sequences sets produced trees with similar precision, the disordered sequence MSAs may be better suited to assessment using phylogeny-based methods. Together, this seems to indicate that how “hard” IDPs are to align and whether or not there is good information for phylogenetic tree construction are not necessarily tightly linked.

### Tree precision and sequence length

One major factor that appears to influence the precision of the phylogenetic trees is the length of the sequences. Theoretically, longer sequences are expected to contain more information, which could better infer a phylogenetic tree. This assumes that more information is either improves or has no impact on the tree construction. Ten out of the 14 proteins produced the most precise trees using whole sequences compared to the ordered and disordered region trees (Fig 4). Additionally, for the mixed proteins, every protein that had ordered region trees that were closer to the species tree than the disordered region trees also had ordered regions that were longer than the disordered regions (Fig 4 and Table 1). Of the nine proteins with disordered region trees that were more precise than the ordered region trees, seven had disordered regions that were longer than their ordered regions. This rule, however, is not absolute. Septin-4 and galectin-3 were the only proteins that had better disordered trees than ordered trees even though their disordered regions were shorter. There were also cases where the disordered and ordered region trees were more precise than the whole sequence trees. Smoothelin-like protein 1 had disordered region trees that were closer to the species tree than the whole sequence tree, while anamorsin, p53, and transcription factor p65 all had ordered region trees that were closer to the species tree than the whole sequence tree.

Sequence length also showed an association with tree distance. Specifically, there was an inverse correlation between the average sequence length and the distance of the phylogenetic tree to the species tree (Figs 5 and 7); the correlations tended to be stronger for the DNA and codon MSAs than the peptide MSAs. Dessimoz and Gil also found that there was a trend between tree precision and MSA length after partitioning eukaryotic sequence data sets by length [17]. However, the pattern they observed was much more prominent in peptide MSAs than nucleotide MSAs, which may be in part due to the diversity of the sequences used. Our sequence sets were limited to *Mammalia*, while Dessimoz and Gil used sequences across Eukaryotes, including *Mammalia*, *Protostomia*, and *Fungi*, but did not explicitly include disordered proteins [17]. If synonymous mutations are assumed to have very little or no impact on fitness, then the DNA/codon sequence can change, while the peptide sequence is still preserved. If a large amount of time has passed, these neutral changes can build up resulting in more divergent sequences regardless of the selection pressures. However, in more recently diverged species where there may be fewer differences in the peptide sequences, codon degeneracy may be beneficial for the additional differences in the nucleotide sequences. This may explain why our best trees came from DNA and codon sequences, whereas Dessimoz and Gil’s best trees came from peptide sequences [17].

For all the DNA and codon MSA correlations between sequence length and tree precision, the disordered sequence’s correlations were weaker than the correlations of the ordered sequences (Figs 5 and 7). Again, this may be due in part to there being more information in the disordered sequences because they are less conserved, so that length is less important. In Table 8, comparisons of fully conserved sites in the MSAs were made to examine the difference between length and sequence variability. Fully conserved sites were selected since they provide no helpful information for inferring phylogenetic trees. Both disordered region MSAs from mixed proteins and fully disordered MSAs had fewer fully conserved sites than the ordered region MSAs and fully ordered MSAs. Therefore, having fewer sites that provide useful information for phylogenetic analysis may increase the strength of the correlation between MSA length and tree precision for the ordered proteins when compared to the disordered proteins, since longer sequences have more potential to contain less conserved sites.

**Table 8:** The averaged fraction of fully conserved sites in the multiple sequence alignments of both disordered and ordered sequences.

	DNA	Codon	Peptide
**Disordered region MSA**	0.10	0.10	0.13
**Ordered region MSA**	0.22	0.21	0.34
**Fully disordered MSA**	0.02	0.02	0.02
**Fully ordered MSA**	0.12	0.12	0.17

### Tree precision and MSA identity

Another factor that influences the MSAs and phylogenetic trees is the identity between the sequences, since the differences and similarities between sequences is exactly what allows us to establish potential evolutionary relationships. From Figs 5 and 7, it was observed that there was a positive correlation between the average pairwise identity and the distance of the phylogenetic tree to the species tree for all peptide MSAs, yet there was no significant correlation for the DNA and codon MSAs. These findings were similar to what Dessimoz and Gil observed [17]. After partitioning their Eukaryotic data sets based on their average divergence for pairs of sequences in PAM units, they found that there was a trend between greater divergence and better tree precision. Similar to our results here, this trend was only observed for the peptide MSAs and not for the nucleotide MSAs. The influence of identity on the precision of the trees from peptide MSAs, but not on the precision of the trees from DNA and codon MSAs, may relate to the “twilight zone”, which refers to the area where the sequence identity of remote homologues and random sequences becomes difficult to separate. In peptide sequences the twilight zone is 20–35% identity [45] whereas in nucleotide sequences the twilight zone is 60–65% identity [46–48]. The twilight zone in peptide sequences occurs at a considerably lower percentage because there are 20 amino acid residues as opposed to 4 nucleotides. This makes it less likely for two unrelated amino acids to align between sequences than two unrelated nucleotides, which could explain why there was no significant correlation for the DNA MSAs [48]. However, this does not explain why there was no significant correlation for the codon MSAs, since they were created by back translating the peptide MSAs. Instead, we speculate that this may be attributed to the alignment of amino acids encoded by different codons, altering the sequence identity.

#### Tree precision and accuracy

The precision of the phylogenetic trees using MSAs were assessed based on their ability to recapitulate the mammalian species tree as a measure of correctness. It is important to re-emphasize that this is a measure of precision, not accuracy. The relationship between all clades in the mammalian species tree have not been fully resolved, and different hypotheses on their divergence have been proposed [18–20]. Therefore, an undisputed species tree that includes all Mammalia does not exist, and any species tree that does include all Mammalia is only a possible representation of the true Mammalia species tree. The TimeTree tree was selected because it is based on trees from almost 4000 studies [19], but since alternative species trees are possible, it is important to consider how these trees may affect the observed results. The species tree used in this study places Perissodactyla and Carnivora in a clade followed by Cetariodactyla [19], while other trees first place Perissodactyla and Cetariodactyla in a clade followed by Carnivora [20]. As an example, the normalized Lee-Ashlock distance between the species tree in Fig 3 and this alternate species tree (S4 Fig) would be 0.080. This distance is relatively small when compared to the distances of the disordered and ordered protein trees to the species tree (Figs 4 and 6), which typically ranged from 0.200 to 0.300. Although using an alternative species tree may result in some changes to the distances measured, we would not expect changes in the overall results when comparing disordered tree precision to ordered tree precision.

Furthermore, two methods were applied to determine the precision of the trees, the Lee-Ashlock and Robinson-Foulds distances. It is worth noting that the Lee-Ashlock distances is relatively unexplored compared the Robinson-Foulds distances, and to our knowledge has not been previously used to perform phylogenetic analyses. Overall, both methods detected the same pattern of no improvement in tree precision for either disordered or ordered sequences (compare Figs 4 and 6 to S1 and S2 Figs). Additionally, when all tree distances were compared, a moderate correlation was observed (S3 Fig). Continued exploration of the Lee-Ashlock space will only improve its utility in comparing trees.

## Conclusion

It has been suggested that IDPs are a challenge to align. While this seems to hold true when comparing the similarity between alignments, trees created from disordered and ordered MSA were not significantly different distances to the species tree, suggesting that the disordered and ordered MSAs are equally sufficient for phylogenetic analysis. Identifying features in the MSA that were correlated to tree precision is challenging, yet the strongest correlation observed was with sequence length. This is unsurprising, as theoretically longer sequences would contain more information that can be used to better resolve the species tree, yet it is not an absolute rule. By better understanding the limitations of alignments and how they affect different data sets we use, we can make steps towards improving how we use these important tools.

## Supporting information

S1 Fig Distance of MrBayes and RAxML consensus trees to the species tree from alignments of whole, ordered region, and disordered region sequences using normalized Robinson-Foulds distances. Circles represent averaged MrBayes consensus tree distances and triangles represent averaged RAxML consensus tree distances. (A) Proteins where the disordered consensus tree is closer to the species tree compared to the ordered consensus tree. (B) Proteins where the ordered consensus tree is closer to the species tree than the disordered consensus tree. Circles represent averaged MrBayes consensus tree distances and triangles represent averaged RAxML consensus tree distances. Orange, whole protein sequence; blue, ordered region sequence; purple, disordered region sequence. *indicates where disordered region trees and ordered region trees were significantly different to each other (*p*-value < 0.05 using Wilcoxon two-sided test).
(EPS)

S2 Fig Distance of MrBayes and RAxML consensus trees to the species tree for all fully disordered and ordered proteins using normalized Robinson-Foulds distances. Ordered proteins (represented by triangles): ▲ alcohol dehydrogenase; ▲ β-galactosidase; ▲ carbonic anhydrase 1; ▲ citrate synthase; ▲ hemoglobin; ▲ hexokinase-1; ▲ insulin; ▲ LDHA; ▲ succinate CoA ligase (ADP forming). Disordered proteins (represented by circles): **●** β-casein; **●** bone sialoprotein; **●** chromogranin-A; **●** ermin; **●** involucrin; **●** osteopontin; **●** protein LBH; **●** prothymosin α; **●** securin.
(EPS)

S3 Fig Correlation between normalized Lee-Ashlock and Robinson-Foulds distance for every tree to the species tree. The Pearson and Kendall R-values are shown in an inset in the upper right corner. *indicates correlation that were significant (*p*-value < 0.05).
(EPS)

S4 Fig Alternate mammal species tree. The tree is based off the species tree in Fig 3, however, Perissodactyla is more closely related to Cetartiodactyla than Carnivora in this tree. Squares represent members of Eurachontoglires, diamonds represent members of Laurasiatherian, and triangles represent all other taxa. The symbol cluster the taxa on orders and superorders; the legend is shown in the figure. Symbols with no colour represent all other taxa.
(EPS)

S1 Table Similarities between the DNA multiple sequence alignments of disordered and ordered regions. The names of the MSA methods indicates the MSAs being compared. The similarities are the average similarity between MSA comparisons swapping the reference and query MSA.
(DOCX)

S2 Table Similarities between the peptide multiple sequence alignments of disordered and ordered regions. The names of the MSA methods indicates the MSAs being compared. The similarities are the average similarity between MSA comparisons swapping the reference and query MSA.
(DOCX)

S3 Table Averaged tree precision for multiple sequence alignments on mixed proteins. * indicates where the tree precision of the disordered region was significantly different from the tree precision of the ordered region (*p*-value < 0.05 using Wilcoxon two-sided test).
(DOCX)
