# Supplementary material for: The difficulty of aligning intrinsically disordered protein sequences as assessed by conservation and phylogeny
Source: PLoS One. 2023 Jul 13;18(7):e0288388. doi: 10.1371/journal.pone.0288388 (PMC10343074; doi:10.1371/journal.pone.0288388)
Supplement: S1 Table — The names of the MSA methods indicates the MSAs being compared. The similarities are the average similarity between MSA comparisons swapping the reference and query MSA. (DOCX) [file pone.0288388.s005.docx]

**Table S1. Similarities between the peptide multiple sequence alignments of disordered and ordered regions.** The names of the MSA methods indicates the MSAs being compared. The similarities are the average similarity between MSA comparisons swapping the reference and query MSA.

| **Proteins** | **Disordered Regions** | | | **Ordered Regions** | | |
| --- | --- | --- | --- | --- | --- | --- |
|  | **Clustal Omega/ MAFFT** | **Clustal Omega/ MUSCLE** | **MAFFT/ MUSCLE** | **Clustal Omega/ MAFFT** | **Clustal Omega/ MUSCLE** | **MAFFT/ MUSCLE** |
| **Anamorsin** | 0.992 | 0.991 | 0.995 | 0.924 | 0.990 | 0.928 |
| **Beclin-1** | 0.866 | 0.875 | 0.972 | 0.960 | 0.962 | 0.983 |
| **Beta-adducin** | 0.971 | 0.970 | 0.991 | 1.000 | 1.000 | 1.000 |
| **DNA topoisomerase 1** | 0.592 | 0.608 | 0.796 | 0.999 | 0.999 | 1.000 |
| **Galectin-3** | 0.430 | 0.425 | 0.489 | 0.982 | 0.982 | 0.996 |
| **Histone H1.0** | 0.941 | 0.940 | 0.984 | 1.000 | 1.000 | 1.000 |
| **Melanophilin** | 0.344 | 0.340 | 0.757 | 0.927 | 0.897 | 0.919 |
| **p53** | 0.608 | 0.624 | 0.760 | 0.991 | 0.992 | 0.999 |
| **Protein Tob1** | 0.805 | 0.769 | 0.857 | 1.000 | 1.000 | 1.000 |
| **Proto-oncogene c-Fos** | 0.935 | 0.935 | 0.967 | 0.998 | 0.998 | 1.000 |
| **Septin-4** | 0.914 | 0.892 | 0.938 | 1.000 | 1.000 | 1.000 |
| **Smoothelin-like protein 1** | 0.498 | 0.515 | 0.621 | 0.893 | 0.891 | 0.996 |
| **Telethonin** | 0.953 | 0.951 | 0.997 | 0.905 | 0.920 | 0.929 |
| **Transcription factor p65** | 0.909 | 0.920 | 0.943 | 0.984 | 0.979 | 0.985 |
