# Supplementary material for: The difficulty of aligning intrinsically disordered protein sequences as assessed by conservation and phylogeny
Source: PLoS One. 2023 Jul 13;18(7):e0288388. doi: 10.1371/journal.pone.0288388 (PMC10343074; doi:10.1371/journal.pone.0288388)
Supplement: S2 Table — The names of the MSA methods indicates the MSAs being compared. The similarities are the average similarity between MSA comparisons swapping the reference and query MSA. (DOCX) [file pone.0288388.s006.docx]

**Table S2. Similarities between the peptide multiple sequence alignments of disordered and ordered regions.** The names of the MSA methods indicates the MSAs being compared. The similarities are the average similarity between MSA comparisons swapping the reference and query MSA.

| **Proteins** | **Disorder** | | | **Order** | | |
| --- | --- | --- | --- | --- | --- | --- |
|  | **Clustal Omega/ MAFFT** | **Clustal Omega/ MUSCLE** | **MAFFT/ MUSCLE** | **Clustal Omega/ MAFFT** | **Clustal Omega/ MUSCLE** | **MAFFT/ MUSCLE** |
| **Anamorsin** | 0.992 | 0.991 | 0.996 | 0.948 | 0.993 | 0.951 |
| **Beclin-1** | 0.925 | 0.957 | 0.955 | 0.973 | 0.971 | 0.974 |
| **Beta-adducin** | 0.971 | 0.960 | 0.967 | 1.000 | 1.000 | 1.000 |
| **DNA topoisomerase 1** | 0.646 | 0.641 | 0.703 | 0.997 | 0.999 | 0.997 |
| **Galectin-3** | 0.538 | 0.503 | 0.549 | 0.985 | 0.985 | 0.997 |
| **Histone H1.0** | 0.941 | 0.941 | 0.999 | 1.000 | 1.000 | 1.000 |
| **Melanophilin** | 0.587 | 0.587 | 0.733 | 0.847 | 0.946 | 0.846 |
| **p53** | 0.659 | 0.635 | 0.680 | 0.984 | 0.984 | 0.990 |
| **Protein Tob1** | 0.794 | 0.800 | 0.846 | 1.000 | 1.000 | 1.000 |
| **Proto-oncogene c-Fos** | 0.943 | 0.946 | 0.972 | 1.000 | 1.000 | 1.000 |
| **Septin-4** | 0.885 | 0.905 | 0.957 | 1.000 | 1.000 | 1.000 |
| **Smoothelin-like protein 1** | 0.613 | 0.610 | 0.706 | 0.999 | 0.999 | 1.000 |
| **Telethonin** | 0.970 | 0.970 | 1.000 | 0.986 | 0.975 | 0.969 |
| **Transcription factor p65** | 0.950 | 0.935 | 0.952 | 0.980 | 0.993 | 0.986 |
