# Supplementary material for: The difficulty of aligning intrinsically disordered protein sequences as assessed by conservation and phylogeny
Source: PLoS One. 2023 Jul 13;18(7):e0288388. doi: 10.1371/journal.pone.0288388 (PMC10343074; doi:10.1371/journal.pone.0288388)
Supplement: S3 Table — * indicates where the tree precision of the disordered region was significantly different from the tree precision of the ordered region (p-value < 0.05 using Wilcoxon two-sided test). (DOCX) [file pone.0288388.s007.docx]

**Table S3. Averaged tree precision for every multiple sequence alignment method used on all mixed proteins.** * indicates where the tree precision of the disordered region was significantly different from the tree precision of the ordered region (*p*-value < 0.05 using Wilcoxon two-sided test). In comparisons of DNA and codon MSAs to peptide MSAs using the same methods, all tree precisions were significantly different (*p*-value < 0.05 using Wilcoxon two-sided test). The only exceptions for the whole sequences were the DNA Clustal Omega RAxML and MrBayes trees. For the disordered sequences the only exceptions were DNA Clustal Omega MrBayes and Codon MUSCLE RAxML trees.

| **MSA method** | **Whole sequence** | **Disordered Region** | **Ordered Region** |
| --- | --- | --- | --- |
| **DNA Clustal Omega RAxML** | 0.331 | 0.411 | 0.429 |
| **DNA Clustal Omega MrBayes** | 0.319 | 0.437 | 0.436 |
| **DNA MAFFT RAxML** | 0.284 | 0.367 | 0.416 |
| **DNA MAFFT MrBayes** | 0.281 | 0.383 | 0.435 |
| **DNA MUSCLE RAxML** | 0.274 | 0.363 | 0.414 |
| **DNA MUSCLE MrBayes** | 0.279 | 0.384 | 0.444 |
| **Codon Clustal Omega RAxML** | 0.307 | 0.376 | 0.419 |
| **Codon Clustal Omega MrBayes** | 0.284 | 0.384 | 0.439 |
| **Codon MAFFT RAxML** | 0.280 | 0.371 | 0.430 |
| **Codon MAFFT MrBayes** | 0.272 | 0.392 | 0.440 |
| **Codon MUSCLE RAxML** | 0.294 | 0.397 | 0.422 |
| **Codon MUSCLE MrBayes** | 0.288 | 0.419 | 0.433 |
| **Peptide Clustal Omega RAxML** | 0.445 | 0.536 | 0.628 |
| **Peptide Clustal Omega MrBayes** | 0.426 | 0.563 | 0.658 |
| **Peptide MAFFT RAxML*** | 0.421 | 0.509 | 0.633 |
| **Peptide MAFFT MrBayes** | 0.430 | 0.550 | 0.662 |
| **Peptide MUSCLE RAxML*** | 0.434 | 0.500 | 0.628 |
| **Peptide MUSCLE MrBayes** | 0.456 | 0.561 | 0.661 |
